# Development and Application of a Cooling Rate Dependent PVT Model for Injection Molding Simulation of Semi Crystalline Thermoplastics

**DOI:** 10.3390/polym16223194

**Published:** 2024-11-17

**Authors:** Thomas Willerer, Thomas Brinkmann, Klaus Drechsler

**Affiliations:** 1 Chair of Carbon Composites, Technical University of Munich, 85748 Garching, Germany; 2 Webasto SE, Kraillinger Straße 5, 82131 Stockdorf, Germany; 3 Department of Plastics Technology, Faculty of Engineering Sciences, Technical University of Applied Sciences Rosenheim, Hochschulstraße 1, 83024 Rosenheim, Germany

**Keywords:** PVT, cooling rate, semi crystalline, thermoplastics, simulation, moldflow, crystallization, injection molding

## Abstract

This technical paper delves into the creation and application of an enhanced mathematical model for semi crystalline thermoplastics based on the Pressure-Volume-Temperature (PVT) Two Domain Tait Equation. The model is designed to incorporate the impact of the cooling rate on the specific volume of the material. This is achieved by utilizing Flash differential scanning calorimetry (fDSC) measurements, thereby ensuring a direct correlation to the actual behavior of the material in reality. The practical application of the model in the context of injection molding simulation was also considered. This was done by integrating the mathematical model into the Moldflow software via the Solver API. The paper underscores the discontinuity issue inherent in the traditional Tait equation with cooling rates and proposes a solution that guarantees a correct transition from the liquid to the solid phase, even at high cooling rates and pressures. The results demonstrated a realistic PVT curve across a wide range of cooling rates and high pressures. The model was put to the test using a 3D tetrahedron meshed calculation model in the injection molding simulation. This study marks a significant step forward in the simulation of injection molding processes, as it successfully bridges the gap between real material properties and simplified simulation, paving the way for more accurate and efficient simulations in the future.

## 1. Introduction

The accuracy of simulations of the injection molding process depends largely on the accuracy of the material models that are used. The description of the PVT (pressure volume temperature) behavior is one of the most important instruments here [1,2]. In order to predict the component properties of plastic products, it is important to include as many phases of plastic injection molding and material behavior as possible [3]. For the simulation, it is therefore important to bring the applied mathematical models and approximations as close as possible to reality. The time-dependent cooling processes, which have a major influence on the specific volume of the plastic material, must also be considered [4,5,6,7,8]. Therefore, a cooling rate-dependent PVT model is to be developed for the first time in this study, which can be implemented in an injection molding simulation program.

The two-domain Tait equation of state model [9,10] currently used in injection molding simulation Moldflow Insight 2023.1 from Autodesk does not take these influences into account. Other models that are used in the injection molding simulation, such as the Spencer Gilmore model [11], which is rarely used due to its simplification, or the Schmidt model [12], also do not take cooling rates into account in their models. The reason for this is that the models describe the PVT behavior in an isothermal or isobar measurement [13] and are not designed to reflect the highly dynamic temperature influences of an injection molding simulation. This should be changed with the help of this work.

There is a mathematical calculation of a cooling rate dependent PVT diagram for amorphous thermoplastics by Chang [14], which has already been transferred to the injection molding simulation software Moldflow from Autodesk for a study [1]. But this mathematical model cannot be used for semi-crystalline thermoplastics. Therefore, in this study, a cooling rate dependent PVT model for semi-crystalline thermoplastics will be developed and compared in the simulation. In order to test new mathematical models from science in the commercially used Moldflow Insight from Autodesk, a solver API interface was implemented by Autodesk, which was used for this work [1]. This interface is also used for this work to integrate the cooling rate dependent on PVT behavior for semi crystalline plastics into the simulation.

The practical application of this model in injection molding simulations represents significant progress. It enables more accurate predictions of material behavior and thus improves the quality and reliability of the manufactured components. This study not only expands the understanding of PVT behavior under varying cooling rates but also paves the way for more efficient and accurate injection molding simulations in the future.

In the Moldflow program from Autodesk, there is already a crystallization kinetic consideration for semi crystalline plastics, which includes the influence of the cooling rate in the crystallization, but this function can only be used for a middle layer or surface meshed parts and is therefore unsuitable for complex geometries [15]. It is known that the crystallinity and therefore the density of semi crystalline thermoplastics are determined by the previous cooling process and the associated cooling rates [4,8,16,17]. In general, the influence of the cooling rate on semi crystalline plastics can be described as follows: As the cooling rate increases, crystallization and density become lower while the specific volume increases, as crystal growth is inhibited by the rapid cooling [7,16]. Meanwhile, the crystallization temperature decreases with increasing cooling rate [18].

PVT measuring instruments cannot currently reproduce the high cooling rates that occur during injection molding [19]. These occur particularly in the edge zone when the component is viewed across the wall thickness, where cooling rates of several hundred to thousand °C/s can occur [1]. The change in crystallinity caused by the high cooling rates can also be influenced by different process controls on the injection molding machine [20]. In order to design the PVT behavior in the injection molding simulation close to the cooling rate, there are already studies that provide a uniform cooling rate over the wall thickness [7]. One possibility is to simply modify the Tait coefficients for that [21], but this is a simplification that does not do justice to the complex geometries of injection-molded parts.

Flash differential scanning calorimetry (fDSC) measurements have been used for a while now to determine the effects of high cooling rates close to the injection molding process on crystallization. These measurements show that the crystallization temperature and crystallinity drop significantly at very high or increasing cooling rates [8,22,23,24]. Filler materials such as the glass fibers used in this study change the onset of crystallization at higher temperatures. However, the crystallinity remains unaffected by the fillers via the cooling rates, as past studies have shown [25].

With the help of the fDSC, the mathematical model in this study can be verified directly with measurements and thus has a direct reference to reality.

## 2. Theory

The PVT behavior can be calculated using the “Two Domain Tait Equations of State”, which will only be called the Tait equation in the following [9,10]. The Tait equation is the most commonly used equation to represent the PVT behavior of thermoplastics [26]. It is used, nevertheless, without taking the cooling rate into account in the simulation [14]. The volume is described by $v\left(p,T\right)$ in (1) [14]:

(1) $$v\left(p,T\right)=v_{0}(p_{0},T)\left\{1-Cln\left[1+\frac{p}{B\left(T\right)}\right]\right\}+v_{t}(p,T)$$

(2) $$T_{t}\left(p\right)=b_{5}+b_{6}p$$

(3) $$\bar{T}=T-b_{5}$$

For $\bar{T}>T_{t}\left(p\right)$:

(4) $$v_{0}(p_{0},T)=b_{1m}+b_{2m}\bar{T}$$

(5) $$B(T)=b_{3m}\;\exp(-b_{4m}\bar{T})$$

(6) $$v_{t}(p,T)=0$$

For $\bar{T}<T_{t}\left(p\right)$:

(7) $$v_{0}(p_{0},T)=b_{1s}+b_{2s}\bar{T}$$

(8) $$v_{0}(p_{0},T)=b_{1s}+b_{2s}\bar{T}$$

(9) $$v_{t}\left(p,T\right)=b_{7}\;\exp(b_{8}\bar{T}-b_{9}p)$$

**Figure 1:**
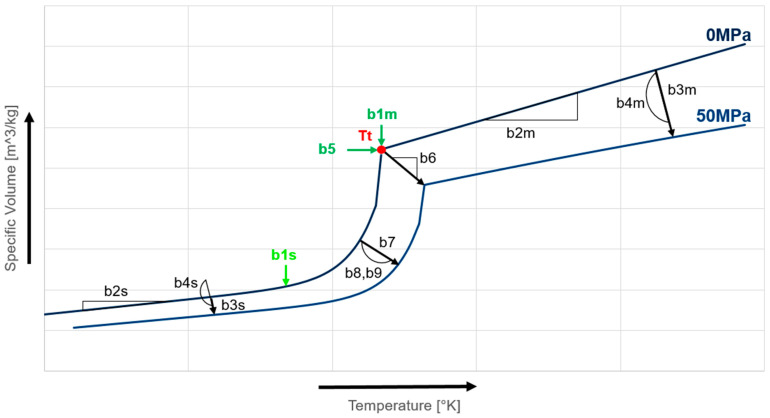
PVT behavior of a semi crystalline plastic with effects of the individual influencing factors of the two-domain Tait equation of state [5].

In this conventional two-domain Tait equation of state (EoS), where C is a universal constant with a value of 0.0894, $v_{0}$ represents the specific volume at zero pressure. The parameters $b_{1}$ ($b_{1m}$, $b_{1s}$) and $b_{2}$ ($b_{2m}$, $b_{2s}$) are employed to articulate the dependency of $v_{0}$ on pressure and temperature. To visualize the influence of these parameters, Figure 1 shows which behavior every factor has. The pressure sensitivity, denoted as B, is a function of temperature alone, determined by two material constants, namely $b_{3}$ ($b_{3m}$, $b_{3s}$) and $b_{4}$ ($b_{4m}$, $b_{4s}$). The specific volume decrease due to crystallization is denoted as $v_{t}$, while $T_{t}$ represents the transition temperature, which separates the two curves of the liquid and solid phases. Parameters $b_{5}$ and $b_{6}$ are utilized to characterize the variation of $T_{t}$ with pressure, whereas $b_{7}$, $b_{8}$, and $b_{9}$ are specific parameters for semicrystalline polymers, describing the form of the state transition. Figure 1 shows the influences of the individual factors on the diagram. It is important to note that the cooling rate is not taken into account in this traditional two-domain Tait EoS [5].

The pressure also had influences on the crystallization [27]. This can also be seen in the PVT measurements and is described by $T_{t}\left(p\right)$ and $v_{t}\left(p,T\right)$, which can be seen in Formula (2). However, the pressure cannot be considered in the flash DSC measurements. Therefore, a simplified approach must be used when including the pressure on the PVT model. The influence of the pressure on the crystallization phase of the PVT behavior at 5 °C/min is in this PVT model also used for the high cooling rates.

There are already models that adjust the factors $b_{5}$ and $b_{1s}$ according to the results of fDSC measurements [8]. Nevertheless, the continuity of the phase transition between liquid and solid on high cooling rates and the adjustment of the $b_{1m}$ factor are not considered in these studies.

## 3. Calculation

For the adapted model, the current cooling rate $\dot{T}=dT/dt$ and the initial cooling rate, ${\dot{T}}_{0}$, are required, which represents the cooling rate during the measured PVT curve. With q from Equation (10), reference can therefore be made to the PVT behavior of the original cooling rate at which the measurements were carried out [5].

(10) $$q=\frac{\dot{T}}{{\dot{T}}_{0}}$$

As described by Pionteck [28], $b_{5}$ represents the beginning of crystallization and thus describes the temperature at which the first molecules form crystals. In order to make $b_{5}$ cooling rate dependent, Wang [5] uses the calculation method of Spina [29], which represents and describes the $b_{1}$ factors according to $b_{1}=b_{11}-b_{12}\times ln(q)$. $b_{5}$ can be seen in Equation (11).

(11) $$b_{5}=b_{51}-b_{52}\times ln(q)$$

For the cooling rate dependent description of $b_{1}$, this model does not use the description of Spina [29], but instead applies our own modifications. Thus, $b_{1m}$ as in the application of crystallization models, the melt phase in the density is not changed by the cooling rate [30].

PVT measurement methods designed and tested for higher cooling rates do not show any shifts in the molten phase [4,31]. This is why the $b_{1m}$ factor only shifts as a function of the start of crystallization with $b_{5}$. This can be seen in Equation (12).

(12) $$b_{1m}=b_{11m}-{((b}_{52}\times \ln(q))\times b_{2m})$$

WAXD (Wide-angle X-ray diffraction) can be used to determine the different crystallization phases, such as alpha, beta, and mesomorph phase, in the material [32]. At the lower cooling rates at which PVT measurements are possible, mainly β phase crystallites are formed [32]. However, this means that the effects of the different phases on the PVT diagram cannot be determined. Therefore, as a simplification, the same influence is assumed at this point via the crystallization level as via the β phase crystallites.

In addition, a further simplified extension of the degree of crystallinity with an increasing cooling rate is carried out, which ignores the mesomorphic phase. This generic crystallization curve without mesomorphic phase is determined in Schawe [24] and is described and evaluated in Piccarolo [33]. However, the relative crystallinity is calculated with a difference to Schawe [24], because q and not the cooling rate itself are used in order to have the reference to the comparative cooling rate of 5 °C/min:

(13) $$X_{c}=1-a_{1}\times \log\left(q\right)-a_{1}\times {(\log\left(q\right))}^{2}$$

Here, $a_{1}$ and $a_{2}$ are used to fit the experimental data. The reason why this simplification can be used for the injection molding simulation for the current stage is that the temperature during the injection molding characteristic cooling process of the entire component is above the recrystallization temperature of the mesomorph phase. This means that the mesomorphic phase would mainly recrystallize. However, it is recommended to simulate the formation and recrystallization of the mesomorphic phase for future considerations, as this process could have an effect on the resulting internal stresses and thus also on the warpage calculation of the injection molding simulation. In this way, $b_{1s}$ is determined as a dependency of $b_{1m}$ and the relative crystallinity $X_{c}$, as follows:

(14) $$b_{1s}=b_{11s}+\left(\left(b_{11m}-b_{11s}\right)\times (1-X_{c})\right)-{((b}_{52}\times ln(q))\times b_{2m})$$

The parameters $b_{2s}$,$\;b_{3s}$, and $b_{4s}$ are also dependent on the degree of crystallization. The correlation can be described here as shown by Baumgärtner [6] using the rule of mixtures. The idea here is that the PVT measurements already provide values for 0% crystallinity in the melt phase. This means that the DSC measurement can be used to calculate the absolute degree of crystallinity at a cooling rate of 5 °C/min, and since the PVT measurement was also taken at 5 °C/min, the values for 100% crystallinity can also be calculated. Schawe [24] describes how the crystallinity is calculated, which is shown in (15). $X_{a}$ stands for the absolute degree of crystallinity and $X_{ref}$ for the reference crystallinity in order to calculate the absolute degree of crystallinity from the relative degree of crystallinity $X_{c}$. However, $X_{ref}$ can also be calculated using the melt enthalpy. Therefore, ${\Delta H}_{m}$ is the melt enthalpy at 100% crystallinity, and the melt enthalpy ΔH is the melt enthalpy measured in the sample. In the Formulas (16) and (17), i=2,3,4 stands for the first numbers of the parameters $b_{2s}$,$\;b_{3s}$ and $b_{4s}$. The parameter $b_{i1s}$ is the parameter that was originally measured in the PVT measurement for the values and $b_{ic}$ stands for the value at 100% crystallinity.

(15) $$X_{ref}=\frac{X_{a}}{X_{c}}=\frac{∆H}{{∆H}_{m}}$$

(16) $$b_{ic}=\frac{b_{i1s}-(1-X_{ref})\times b_{im}}{X_{ref}}$$

(17) $$b_{is}=X_{a}\left(b_{ic}-b_{im}\right)+b_{im}$$

Wang [5,34] presents a solution for the discontinuity problem of the two-domain EoS, which represents a continuous transition from the liquid to the solid phase even at high cooling rates.

$T_{t}\left(p\right)$ is considered for 0 MPa, i.e., $T_{t}=b_{5}$ where ${T=T}_{t}$ results in $\bar{T}=T-T_{t}=0$. With these conditions, the volume $v_{m}$ for the molten state and $v_{s}$ for the solid state at $T_{t}$ can now be set equal.

(18) $$v_{m}=b_{1m}\times \left[1-C\times \ln\left(1+\frac{p}{b_{3m}}\right)\right]=v_{s}$$

(19) $$=b_{1s}\times \left[1-C\times \ln\left(1+\frac{p}{b_{3s}}\right)\right]+b_{7}\times \exp(-b_{9}\times p)$$

(20) $$b_{7}\times \exp(-b_{9}\times p)=b_{1m}\times \left[1-C\times \ln\left(1+\frac{p}{b_{3m}}\right)\right]-b_{1s}\times \left[1-C\times \ln\left(1+\frac{p}{b_{3s}}\right)\right]$$

Equation (20) is now inserted into (9) and thus results in $\bar{T}<T_{t}\left(p\right)$:

(21) $$v_{t}\left(p,T\right)=exp(b_{8}\bar{T})\times \left\{b_{1m}\times \left[1-C\times \ln\left(1+\frac{p}{b_{3m}}\right)\right]-b_{1s}\times \left[1-C\times \ln\left(1+\frac{p}{b_{3s}}\right)\right]\right\}$$

However, this continuity condition of Wang [5,31] is only valid for 0 MPa, which is why a correction is made for higher pressures on the basis of $b_{3m}$:

(22) $$b_{3m}=b_{31m}+b_{32m}\times ln\left(q\right)-b_{33m}\times p$$

Here, $b_{31m}$, $b_{32m}$ and $b_{33m}$ are adjusted in such a way that they ensure continuity and reflect the measured values over the entire pressure curve during the changes of the crystallization and in the melt phase. This is necessary because otherwise the modifications from (18)–(21) will result in the pressure no longer having the same influence on the calculation. Thus (22) is needed to do justice to the original PVT measurements. 

## 4. Experiments

**Figure 2:**
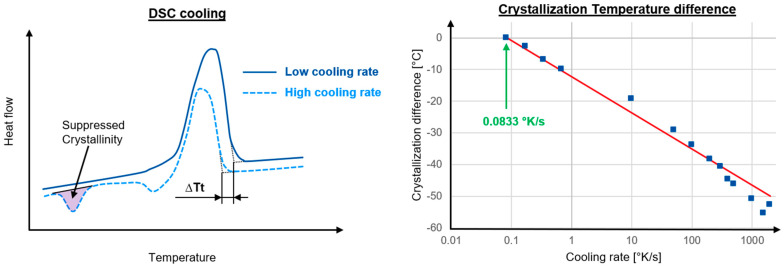
(**left**) Schematic cooling run of the DSC measurement with different cooling rates; (**right**) Crystallization temperature difference over the cooling rates with the new $b_{5}$ coefficients in red.

For this study, the material polypropylene with 40% fiber weight content is called GFPP-40 HSBK126 from KINGFA CI. & TECH. (Europe) GmbH, Wiesbaden, Germany, is used. Cooling rate dependent crystallization was already investigated 30 years ago using DSC measurements [35]. As seen in Zhuravlev [18] and Schawe [24], very high constant cooling rates of 10–2000 °C/s are mapped with the flash DSC (fDSC) measurement. The measuring device used was a Flash DSC 1 chip calorimeter from Mettler Toledo, Columbus, OH, USA, in which the measurements were carried out in a nitrogen atmosphere at a temperature range from 260 °C to −60 °C. The subsequent heating measurement was also performed in a nitrogen atmosphere. The subsequent heating measurement has been analyzed at a heating rate of 200 K/s. The low cooling rates of 5–40 °C/min were carried out with a normal DSC Discovery 2500 measuring device from TA Instruments, Eschborn, Germany, in a nitrogen atmosphere in a temperature range of −20 to 220 °C. The heating rate was 10 K/min. The subsequent heating measurements are used to measure the degree of crystallization. Just as peak crystallization temperatures can be determined via the cooling rate [24,36], the temperature at the start of crystallization can also be determined via the cooling rate in accordance with ISO 11357-1:2023 [37]. The resulting difference between the high and low cooling rates can thus be read from the DSC curves, as shown in Figure 2 on the left, and thus the crystallization temperature difference $∆T_{t}$ can be determined. As already described, the cooling rate of 0.0833 °K/s is used as a reference, and the resulting differences of the other cooling rates are plotted to the right in Figure 2. The red line indicates the crystallization temperature difference $b_{5}$, when $b_{52}=4.95$.

**Figure 3:**
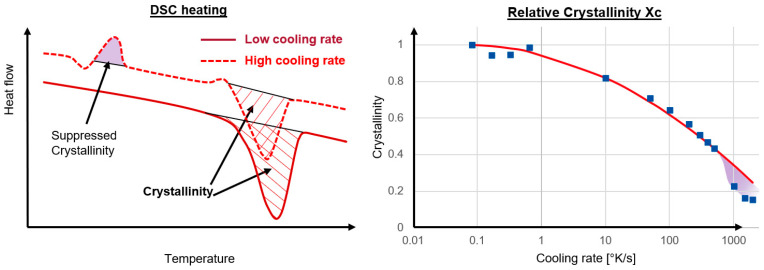
(**left**) Schematic heating run after the DSC measurement with different cooling rates; (**right**) Crystallization difference over the Cooling rates with calculated $X_{c}$ in red.

In the subsequent heating curve of the fDSC, even at very low temperatures of 10–20 °C, a deflection can be seen in the diagram, which represents the recrystallization of the mesomorphic phase [38,39]. To show this phenomenon, the suppressed crystallinity in the schematic fDSC curve of the high cooling rate is shown on the left in Figure 3. At a heating rate of 1000 K/s, the melting of the mesophase can still be seen very well in the DSC measurements, whereas at lower heating rates the melting of the mesophase already takes place at lower temperatures [38]. As with the crystallization temperature, the cooling rate of 0.0833 °K/s is used as a reference, and the resulting crystallization difference of the other cooling rates is plotted on the right in Figure 3. The red line denotes the crystallization $X_{c}$ from (13).

The values for the absolute degree of crystallinity $X_{a}$ can also be determined from the crystallization measurements. For the cooling rate of 0.0833 °K/s, a melt enthalpy ∆H of 100.24 J/g without glass fiber was determined in the DSC measurement. These results, combined with the literature value ${∆H}_{m}$ = 205 J/g from Ehrenstein [13] with (15), in $X_{ref}$ = 0.489.

An Isobaric measurement method with a cooling rate of 5 °C/min is used to determine the PVT data to be able to draw direct conclusions from the DSC measurement. The PVT measuring device PVT 500 from Göttfert is used for this purpose. The pressures measured are 2000, 1500, 1000, and 500 bar. In order to show the PVT properties at 0 bar, the results were extrapolated and the coefficients of the Tait model were determined. The Tait coefficients are shown in Table 1. The cooling rate dependent coefficients for the new method are shown in the second column, while the standard coefficients are shown in the first column.

Currently in most simulation software for injection molding, a solidification temperature is also required here, at which the calculation changes from the liquid to the solid phase and from the isotropic calculation to an anisotropic calculation. This temperature depends on the degree of crystallinity [40,41]. By implementing the cooling rate in the PVT calculation, this temperature also changes, as the crystallization temperature and the degree of crystallization change, as already described [42]. Since this study deals with the shrinkage properties and not the warpage, the solidification temperature is set to the temperature of $b_{1s}$ in order not to switch too early in the anisotropic thermal expansion. This is because these are not temperature- or crystallization-dependent on this standard Simulation model. It should be noted that therefore the warpage of the component cannot be determined, only the shrinkage.

## 5. Results

**Figure 4:**
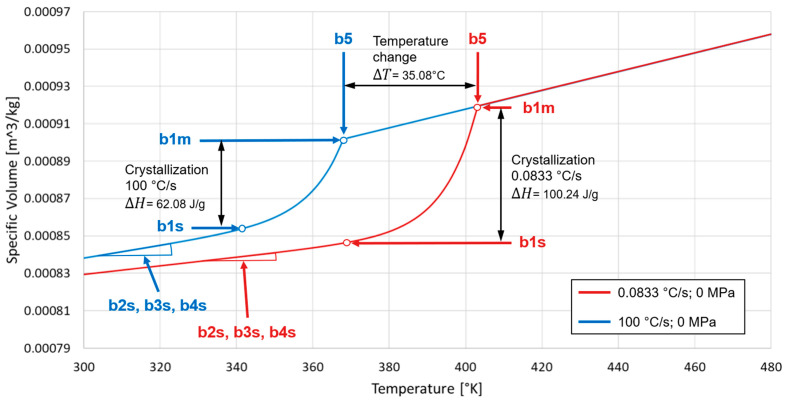
PVT diagram with the influence of the cooling rate.

Since, as already mentioned, the high cooling rates cannot be reproduced in the PVT measurements [19], only the calculated PVT diagrams can be compared with each other, which have a connection to the real PVT properties using the fDSC measurement. To explain the influences of the newly introduced coefficients in more detail, Figure 4 shows how the coefficients and the new calculation method affect the change from the diagram between a cooling rate of 0.0833 °C/s and 100 °C/s. At this point, it should be mentioned that the diagram at 0.0833 °C/s shows the same curve as the standard Tait equation. Ultimately, this mathematical method only changes the parameters that can be verified from the fDSC measurements. As shown in Figure 4, the crystallization temperature $b_{5}$ is lower with an increasing cooling rate, analogous to the fDSC measurement in Figure 2. This also applies to the degree of crystallization, which is described between $b_{1m}$ and $b_{1s}$ and is determined by $b_{1s}$ in the mathematical model. The coefficients $b_{2s}$, $b_{3s}$, and $b_{4s}$ change depending on the degree of crystallization. 

**Figure 5:**
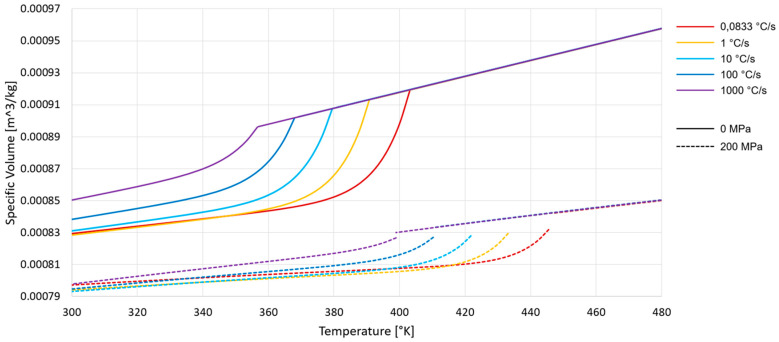
PVT diagram with different cooling rates in 0 MPa and 200 MPa.

To show that the new mathematical PVT model works from the minimum cooling rates of 0.0833 °C/s up to at least 1000 °C/s, the cooling rates are shown in gradations in Figure 5. This figure also shows that by modifying the $b_{3m}$ factor at 0 MPa and also at 200 MPa, no anomalies can be recognized.

**Figure 6:**
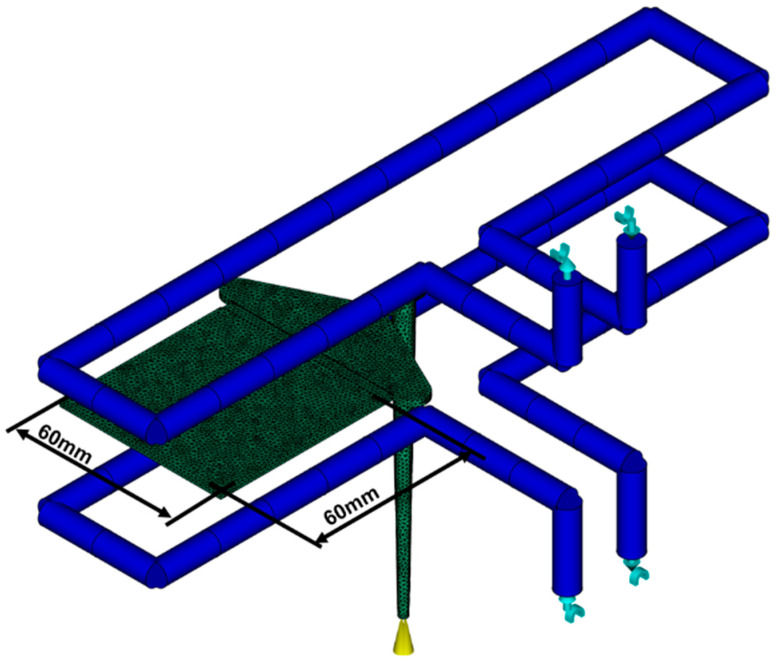
3D meshed geometry with injection point and cooling channels in the tool.

To prove that the new PVT model can be transferred to an injection molding simulation, the new mathematical PVT model is transferred to Moldflow via the solver API described above. A 3D tetrahedron meshed calculation model with 20 layers over the 2 mm thick wall thickness is used as a test model, which can be seen in Figure 6. The model is derived from a real test mold and represents a plate, which is connected to the gating system via a tie bar. The plate has a length of 60 mm and a width of 60 mm. All results generated in this work, which were taken at one point and show the result of the wall thickness, were taken from the center of this plate.

**Figure 7:**
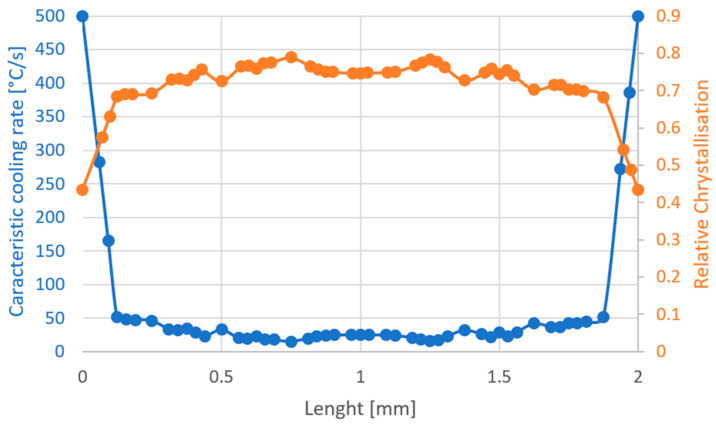
(Blue) Characteristic cooling rate through thickness and (Orange) Relative crystallization through thickness.

In order to show what influence the plastic injection molding process has on the material and the time-temperature processes, the characteristic cooling rate is determined, as in Cook [1], at which the PVT calculation has its transition point and switches from the liquid to the solid calculation formulas. From the point of view of the mathematical model, this is the cooling rate that occurs at $T_{t}\left(p\right)$. This characteristic cooling rate together with the resulting relative crystallinity $X_{c}$ is shown in the diagram in Figure 7. In the calculation solver, the characteristic cooling rate is determined as soon as $T_{t}\left(p\right)$ is reached. The following calculations concerning the crystallization are carried out with this characteristic cooling rate. A maximum of 500 °C/s has been set for the cooling rate in the simulation.

An important result of the calculation resulting from the PVT information is the volume shrinkage. According to Cook [1], the volume shrinkage is determined at each node from the percentage difference between a reference-specific volume and the specific volume at the point in time at which the local flow stops. This is the case as soon as the pressure drops to zero or the material reaches the solidification temperature. This is the conclusion from Cook [1]:

(23) $$S_{\left(node\right)}=\frac{v\left(T_{node},p_{node},{\dot{T}}_{node}\right)-v\left(T_{amb},0,{\dot{T}}_{node}\right)}{v\left(T_{amb},0,{\dot{T}}_{node}\right)}$$

**Figure 8:**
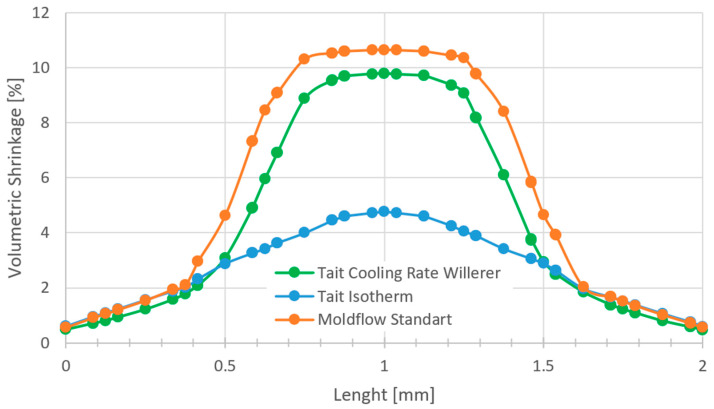
Volumetric shrinkage through thickness.

$T_{amb}$ stands for the room temperature. In order to compare the result of the volume shrinkage with the state of the art, the standard calculation of the Moldflow software and the classic Tait PVT model are compared with the isothermally measured PVT data stored in Moldflow, which is also used for the standard Moldflow calculation. This comparison over the part thickness in the middle of the probe plate is illustrated in Figure 8.

A lower volume shrinkage can be observed in the edge area in the first 0.4–0.5 mm, while in the middle the cooling rate dependent calculation, just like the standard Moldflow calculation, shows a significantly higher shrinkage compared to the classic Tait model.

## 6. Conclusions

In this work, a new mathematical PVT model for semi crystalline thermoplastics was developed and successfully integrated into the injection molding simulation software Moldflow. This model considers the dependence of the specific volume on the cooling rate based on real measurements. The fDSC measurements carried out have shown that both the crystallization temperature and the degree of crystallization are significantly influenced by the cooling rate. These influences were mapped in the new model by adapting the Tait equation and introducing cooling rate dependent coefficients.

It should be particularly emphasized that the continuity of the phase transition between liquid and solid phases, which was problematic in the traditional Tait equation, was ensured in the new model by the method proposed by Wang [34]. With the new model presented, a realistic PVT curve can now be guaranteed even at high pressures and a wide range of cooling rates.

For future work, it is recommended to investigate the occurrence and effect of the mesomorphic phase on real components in more detail. In addition, flow-induced crystallization, which also has an influence on PVT behavior during injection molding, was not addressed [43,44]. The influence of pressure on crystallization at different cooling rates should also be investigated in more detail. This was not done in this work, as the current measurement methods such as high-pressure DSC have a cooling rate range that is too small to collect relevant data for injection molding [45] or the fDSC measurement method does not allow measurement under pressure [18].

If this work is used to predict the warpage of injection-molded components, it must be ensured that the solidification temperature, at which the shrinkage calculation changes from isotropic to anisotropic due to the fiber reinforcement, must be examined more closely. In addition, the CTE value and the modulus of elasticity must also be included in the simulation depending on the temperature and cooling rate, which is currently not the case. The development and implementation of a cooling rate-dependent PVT diagram, which can be implemented in the simulation program, is therefore only the first important step towards obtaining reliable simulation data in the future.

Overall, this work represents a significant advance in the simulation of injection molding processes by further closing the gap between real material properties and simplified simulation using experimental measurements.

## Figures and Tables

**Table 1:** Two-domain Tait coefficients.

Old Coefficient	New Coefficient	Value	Unit
$b_{1m}$	$b_{11m}$	9.194 × 10^−4^	m^3^/kg
$b_{2m}$	$b_{2m}$	4.999 × 10^−7^	m^3^/(kg× K)
$b_{3m}$	$b_{31m}$	1.033 × 10^8^	Pa
$b_{4m}$	$b_{4m}$	3.431 × 10^−3^	1/K
$b_{1s}$	$b_{11s}$	8.531 × 10^−4^	m^3^/kg
$b_{2s}$	$b_{21s}$	2.297 × 10^−7^	m^3^/(kg× K)
$b_{3s}$	$b_{31s}$	2.376 × 10^8^	Pa
$b_{4s}$	$b_{41s}$	3.878 × 10^−3^	1/K
$b_{5}$	$b_{51}$	4.0315 × 10^2^	K
$b_{6}$	$b_{6}$	2.115 × 10^−7^	K/Pa
$b_{7}$		5.456 × 10^−5^	m^3^/kg
$b_{8}$	$b_{8}$	1.156 × 10^−1^	1/K
$b_{9}$	$b_{9}$	2.689 × 10^−8^	1/Pa
	$b_{52}$	4.95	°K
	$X_{ref}$	0.489	
	$a_{1}$	0.01	
	$a_{2}$	0.037	
	$b_{32m}$	2.90 × 10^6^	Pa
	$b_{33m}$	1.30 × 10^−1^	

## Data Availability

The original contributions presented in the study are included in the article, further inquiries can be directed to the corresponding author.
